# Long-Term Tacrolimus Blood Trough Level and Patient Survival in Adult Liver Transplantation

**DOI:** 10.3390/jpm11020090

**Published:** 2021-02-01

**Authors:** Chih-Yang Hsiao, Ming-Chih Ho, Cheng-Maw Ho, Yao-Ming Wu, Po-Huang Lee, Rey-Heng Hu

**Affiliations:** 1 Graduate Institute of Clinical Medicine, College of Medicine, National Taiwan University, Taipei City 110, Taiwan; cyhsiao1102@gmail.com; 2 Department of Surgery, National Taiwan University College of Medicine, Taipei City 110, Taiwan; mcho1215@ntu.edu.tw (M.-C.H.); wyaoming@gmail.com (Y.-M.W.); 3 Department of Surgery, National Taiwan University Hospital, Taipei City 110, Taiwan; miningho@ntu.edu.tw (C.-M.H.); pohuang1115@ntu.edu.tw (P.-H.L.); 4 Department of Traumatology, National Taiwan University Hospital, Taipei City 110, Taiwan; 5 Department of Surgery, E-Da Hospital, I-Shou University, Kaohsiung 886, Taiwan

**Keywords:** Cox’s model, immunosuppressant, liver transplantation, survival, tacrolimus

## Abstract

Tacrolimus is the most widely used immunosuppressant in liver transplant (LT) patients. However, the ideal long-term target level for these patients is unknown. This retrospective study aimed to investigate the impact of tacrolimus blood concentration five years after LT on long-term patient survival outcomes in adult LT recipients. Patients who underwent LT between January 2004 and July 2014 at a tertiary medical center were included in this study (*n* = 189). The mean tacrolimus blood concentrations of each patient during the fifth year after LT were recorded and the overall survival rate was determined. A multivariate analysis of factors associated with long-term survival was conducted using a Cox’s model. The median follow-up period was 9.63 years, and 144 patients (76.2%) underwent live donor LT. Sixteen patients died within 5 years of LT. In the Cox’s model, patients with a mean tacrolimus blood trough level of 4.6–10.2 ng/mL had significantly better long-term survival than those with a mean tacrolimus blood trough level outside this range (estimated hazard ratio = 4.76; 95% confidence interval: 1.34–16.9, *p* = 0.016). Therefore, a tacrolimus level no lower than 4.6 ng/mL would be recommended in adult LT patients.

## 1. Introduction

Liver transplantation (LT) has become a mature treatment of end-stage liver disease in clinical practice [1]. The continuous improvement of effective immunosuppression treatments has led to a significant improvement in patient and graft survival in recent years [2]. Calcineurin inhibitors are the cornerstone of immunosuppression in LT [3], and tacrolimus is currently the mainstay of initial and maintenance immunosuppression therapies [4,5,6]. Tacrolimus reduces the incidence and severity of early and late T-cell mediated rejection by inhibiting T-cell production of interleukin-2 [5,6]. However, the long-term use of immunosuppressants leads to an increasing burden of toxicity. The reported toxic effects of calcineurin inhibitors include infections, chronic renal insufficiency, metabolic diseases (such as hyperlipidemia, hypertension, and diabetes mellitus), and malignancy [7,8,9], which, along with cardiovascular diseases, have been reported to be the major causes of morbidity and mortality after LT [3,10]. However, most LT patients cannot withdraw from lifelong immunosuppression therapy; the only exceptions are a few selected patients participating in experimental trials [11,12].

It is a clinical challenge to design a well-balanced immunosuppressive regimen for LT recipients. Tacrolimus has a narrow therapeutic dose range and its blood levels should be carefully monitored. The Advagraf (tacrolimus prolonged-release hard capsules) recommendation for adult LT patients is a blood trough level of 5–20 ng/mL in the early post-transplant period and 5–15 ng/mL during subsequent maintenance therapy. Nevertheless, the current recommendations from the clinical practice guidelines for the ideal tacrolimus level in adults after LT remain controversial. The American Association for the Study of Liver Diseases (AASLD) recommends a target blood trough level of 5–10 ng/mL for tacrolimus three months after LT [13]. The Consensus on Managing Modifiable Risk in Transplantation Group (COMMIT) recommends that the target tacrolimus blood trough levels be 6–10 ng/mL during the first month after LT and decrease to 4–8 ng/mL thereafter, except when used in combination with mammalian target of rapamycin (mTOR) inhibitors [14]. Moreover, the International Liver Transplant Society (ILTS) consensus statement on immunosuppression in LT recipients recommends the target blood trough levels of tacrolimus be 6–10 ng/mL three months after LT, lower than 5 ng/mL 12 months after LT, and decrease to 3 ng/mL thereafter, resulting in a blood trough level just above the lower limit of detection five years after LT [15]. However, the impact of the long-term tacrolimus blood trough level on the outcomes of the LT recipients remains unclear. This study aimed to suggest an appropriate tacrolimus blood trough level for adult patients five years after LT.

## 2. Materials and Methods

**Figure 1:**
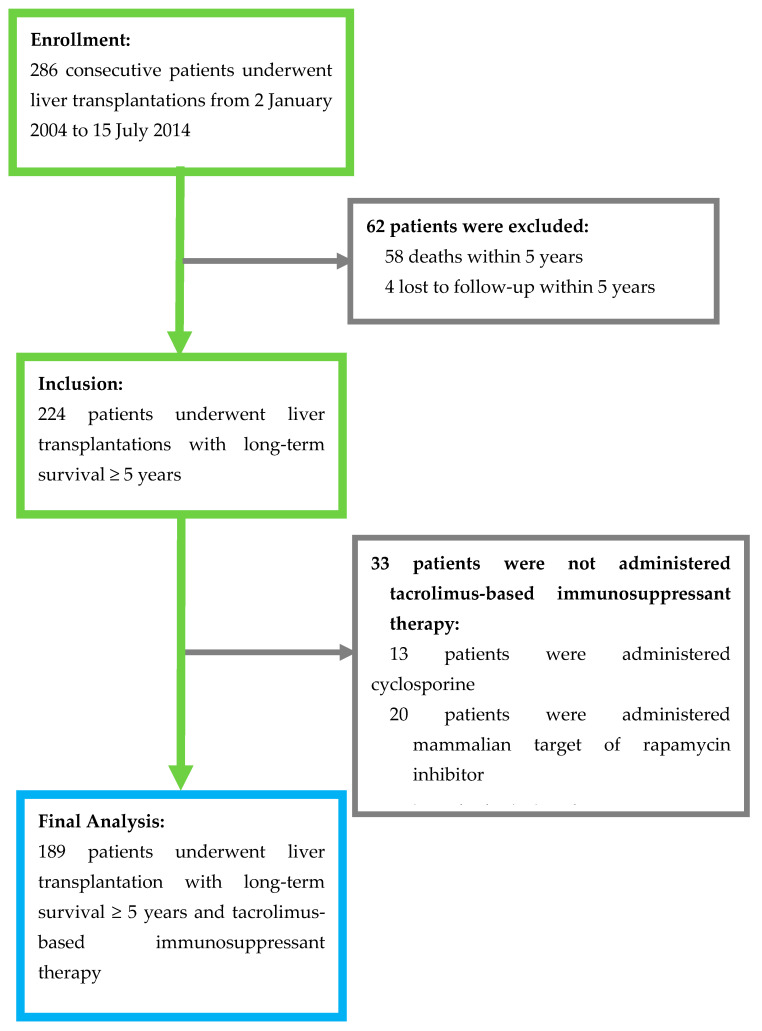
Patient flow diagram.

This study was approved by the Institutional Review Board of National Taiwan University Hospital and was conducted according to the Declaration of Helsinki. A total of 286 patients who underwent LT at 18 years or older at a tertiary medical center from January 2004 to July 2014 were recruited for this study. Patients who died within 5 years of LT, were lost to follow-up, or did not use tacrolimus-based calcineurin inhibitor for immunosuppressant therapy were excluded from this study (Figure 1). In addition, those who received mTOR inhibitor treatment were excluded, as mTOR inhibitors are typically used as a combination therapy to reduce the required dose of tacrolimus. The final analysis included 189 patients. All eligible patients were followed up for more than five years until August 2019. Patients who underwent a LT due to liver cancer met the Milan criteria (before 2006) or the criteria of the University of California, San Francisco (since 2006) at the time of LT. All patients received regular monthly or bi-monthly follow-ups at the outpatient clinic after LT. Routine blood examinations for the tacrolimus blood trough level, liver function, and renal function were conducted at each visit, and abdominal sonography was performed every 6 to 12 months.

The patients’ medical records were reviewed retrospectively to extract demographic and clinical data, including patient characteristics, laboratory tests, and survival outcomes. The serum bilirubin and creatinine data at the end of the fifth year after LT were used in this study. The tacrolimus level used in this study was the mean of the values obtained at the three follow-up visits during the fifth year after LT.

The immunosuppression protocol for adult LT patients consisted of tacrolimus, mycophenolate mofetil, and steroids. Tacrolimus was administered orally beginning on the first day after LT, and the dose was adjusted to achieve the desired therapeutic drug level. Basiliximab was administered immediately before graft reperfusion and on the fourth day after LT for induction therapy. A 500-mg intravenous bolus of methylprednisolone was administered immediately before reperfusion of the liver graft and was tapered to oral prednisolone over one week and reduced to withdrawal after six months.

All statistical analyses were performed using R 4.0.2 software (R Foundation for Statistical Computing, Vienna, Austria). Statistical significance was set at *p* ≤ 0.05. Continuous variables are presented as mean ± standard deviation (SD) and median (interquartile range, IQR), and categorical variables are presented as frequency (percentage, %). The survival curve was estimated by the Kaplan-Meier method. A univariate analysis was conducted to examine the differences in the distributions of continuous variables, categorical variables, and survival outcomes between the surviving and deceased liver recipients five years after LT using the Wilcoxon rank-sum test, Chi-square test, Fisher’s exact test, or log-rank test as appropriate for the data type. A multivariate analysis was performed to estimate the adjusted effects of risk factors or prognostic factors on the survival outcome using a multiple Cox’s proportional hazards model.

To ensure a good quality of regression analysis, the model-fitting techniques for variable selection, goodness-of-fit (GOF) assessment, and regression diagnostics and remedies were used in our regression analysis. Specifically, the stepwise variable selection procedure (with iterations between the forward and backward steps) was applied to obtain the best final regression model using the My.stepwise package of the R software [16]. All significant and non-significant relevant covariates from the univariate analysis (listed in Table 1) and some of the interaction terms were used in the multivariate analysis. The significance levels for entry and for stay were set to 0.15 for being conservative. With the aid of substantive knowledge, the best candidate final regression model was identified manually by dropping the covariates with *p* value > 0.05 one at a time until all regression coefficients were significantly different from 0. To assess the GOF of the fitted Cox’s model, the concordance and adjusted generalized *R*^2^ [17] were examined. A concordance ≥ 0.7 and an adjusted generalized *R*^2^ > 0.15 indicated an acceptable level of discrimination, power, and fitness.

Moreover, the smoothing option “pspline” (for the smoothing splines using a “p-spline” basis) was specified inside the coxph function of the survival package to smooth the effects of continuous covariates on the log-hazard rate of the simple and multiple Cox’s proportional hazards models in R. Then, the termplot function of the stats package was used to plot the smoothed effects of the continuous covariates on the log-hazard rate in R [18]. The regression diagnostics for the verification of proportional hazards assumption, residual analysis, detection of influential cases, and a multicollinearity check were applied to discover any model or data problems. A variance inflating factor (VIF) ≥ 10 in continuous covariates or VIF ≥ 2.5 in categorical covariates indicated the occurrence of the multicollinearity problem among some of the covariates in the fitted regression model.

## 3. Results

### 3.1. Patients’ Demographic and Clinical Characteristics

**Figure 2:**
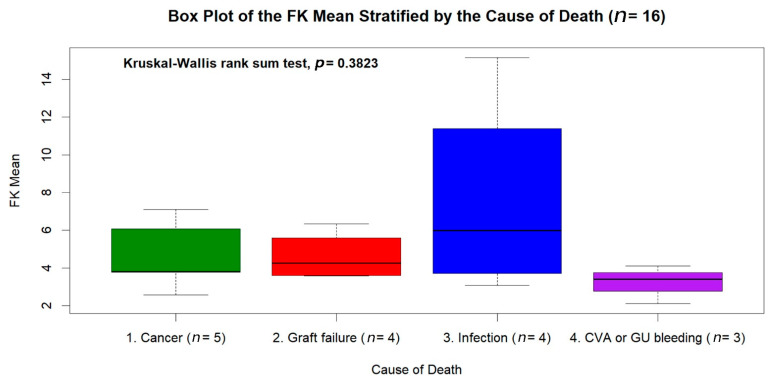
The box plot of tacrolimus mean trough level stratified by the etiology and cause of death among the 16 dead patients. There was no statistical difference of tacrolimus level between the different causes of death (*p* = 0.3823).

The median follow-up duration was 9.63 years (IQR: 7.2–11.4 years), and the mean follow-up was 9.58 ± 2.74 years. A total of 121 males (64.0%) and 68 females (36.0%) were included in this study (Table 1). The median age at LT was 54.26 years (IQR: 48.63–58.58 years, range: 18.3–73.1 years), and the mean age at LT was 52.7 ± 9.6 years. Among the 189 patients, 114 (76.2%) underwent living donor transplants and 45 (23.8%) underwent deceased donor transplants. The main indications for LT were hepatitis B virus (HBV) cirrhosis (52.9%), hepatocellular carcinoma (38.1%), hepatitis C virus (HCV) cirrhosis (22.2%), and fulminant hepatitis (12.2%). The median of the mean tacrolimus blood trough level during the fifth year after LT was 5.0 ng/mL (IQR: 4.12–6.33 ng/mL), and the mean tacrolimus blood trough level was 5.249 ± 1.71 ng/mL. The mean tacrolimus blood trough level was ≥ 5 ng/mL in 96 patients (50.8%) and < 5 ng/mL in 93 patients (49.2%), including 44 patients (23.3%) with a mean tacrolimus blood trough level < 4 ng/mL and 11 patients (5.8%) with a mean tacrolimus blood trough level < 3 ng/mL. No significant differences in gender, age at LT, body weight, blood type, graft type, etiology of LT, or serum total bilirubin were found between the deceased and surviving LT recipients (*p* > 0.05). However, serum creatinine levels > 1.5 mg/dL (23/173 vs. 8/16, *p* = 0.001), end-stage renal disease (2/173 vs. 3/16, *p* = 0.005), and mean tacrolimus blood trough levels < 4 ng/mL (36/173 vs. 8/16, *p* = 0.014) were significantly more common among deceased LT recipients compared to survivors. The causes of death included malignancies (*n* = 5), graft failures (*n* = 4), infection (*n* = 4), cerebrovascular accidents (*n* = 2), and duodenal ulcer bleeding (*n* = 1) (Table 2). The distribution of the tacrolimus trough level stratified by the etiology and cause of death among the 16 dead patients was shown in the box plot of Figure 2. There was no statistical significance in the tacrolimus trough level between the different causes of death (*p* = 0.3823).

### 3.2. Predictors of Patients’ Long-Term Survival

The Cox’s model fitted to the survival data for the multivariate analyses of the time to death after five years of LT is shown in Table 3. After adjusting for the effects of the other covariates, age at LT ≤ 27.011 years (estimated hazard ratio [HR] = 168.79, 95% confidence interval [C.I.]: 11.13–2559.51), pre-transplant autoimmune liver disease (HR = 8.12, 95% C.I.: 1.97–33.43), pre-transplant HCV infection × survival time in years (HR = 1.34, 95% C.I.: 1.12–1.60), serum creatinine level > 1.311 mg/dL × serum total bilirubin level > 1.411 mg/dL (HR = 921.69, 95% C.I.: 43.40–19,573.71), serum creatinine level > 1.311 mg/dL × serum total bilirubin level ≤ 0.792 mg/dL (HR = 105.68, 95% C.I.: 7.81–1430.79), serum creatinine level ≤ 1.311 mg/dL × serum total bilirubin level > 0.882 mg/dL (HR = 30.49, 95% C.I.: 2.98–312.34), and the mean tacrolimus trough level during the fifth year after LT ≤ 4.609 ng/mL or > 10.168 ng/mL (HR = 4.76, 95% C.I.: 1.34–16.94) were associated with a higher long-term mortality five years after LT. The time-dependent interaction term, HCV × survival time in years, was added to the Cox’s model to account for the non-proportional hazards problem between the patients with and without HCV, and its positive-valued regression coefficient estimate, 0.292, indicates that the risk of mortality in patients with HCV increases with time five years post-LT. Moreover, the three second-order interaction terms between the serum creatinine and total bilirubin levels were compared to the other two possible combinations, serum creatinine level > 1.311 mg/dL × (0.792 mg/dL < serum total bilirubin level ≤ 1.411 mg/dL) and serum creatinine level ≤ 1.311 mg/dL × serum total bilirubin level ≤ 0.882 mg/dL, as the reference group (i.e., HR = 1.0), where the cross sign × can be literally interpreted as “and”.

**Figure 3:**
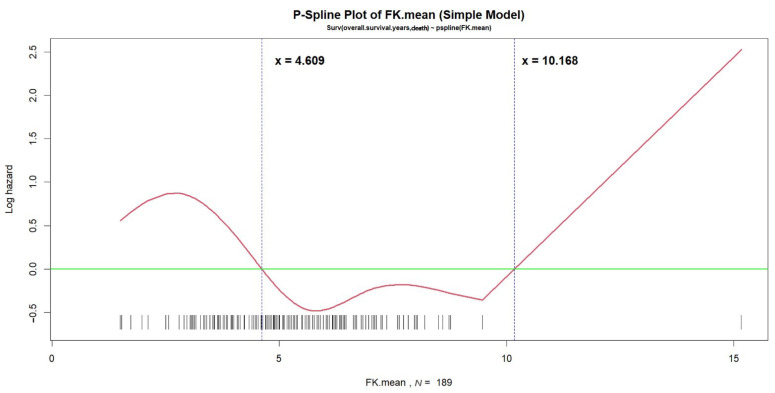
The p-spline plot for the nonlinear effect of the long-term tacrolimus blood trough levels on time to death. The tacrolimus blood trough level is shown on the *X*-axis, and the log(*λ*), where *λ* is the hazard rate of time to death five years after liver transplantation, is shown on the *Y*-axis. In this p-spline plot, the intersection between the horizontal green line (*Y* = 0) and the red curve yields the estimated optimal cut-off values for long-term tacrolimus blood trough levels at which the values of log(*λ*) will not change (4.609 ng/mL and 10.168 ng/mL). When the level is ≤4.609 ng/mL or >10.168 ng/mL, the value of log(*λ*) increases, indicating an increasing *λ.* When the level is >4.609 ng/mL and ≤10.168 ng/mL, the value of log(*λ*) decreases, indicating a decreasing *λ.* The vertical bars above the *X*-axis represent the patients’ actual mean tacrolimus blood trough levels obtained at the three follow-up visits during the fifth year after LT.

**Figure 4:**
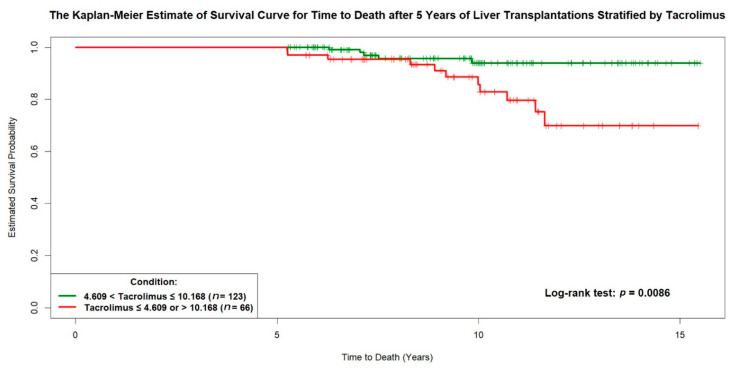
The Kaplan-Meier curve for time to death five years after liver transplantation. The survival curve of the 66 patients with a mean tacrolimus blood trough level ≤ 4.609 ng/mL or >10.168 ng/mL is shown in red while the survival curve of the 123 patients with a mean tacrolimus blood trough level > 4.609 ng/mL or ≤10.168 ng/mL is shown in green.

All cut-off values of the continuous covariates (such as age at LT) were estimated by applying the p-spline smoothing techniques in fitting simple and multiple Cox’s proportional hazards models. As shown in Figure 3, the optimal cut-off values of the mean tacrolimus trough level, 4.609 ng/mL and 10.168 ng/mL, were estimated directly in the “p-spline plot,” which allowed the visualization of the nonlinear effect of the averaged dosage of the tacrolimus-based immunosuppressant during the fifth year after LT on log(*λ*), where *λ* was the hazard rate of time to death five years after LT. Then, the Kaplan-Meier estimates of survival curves for time to death five years after LT were determined for the 66 patients with mean tacrolimus trough levels ≤ 4.609 ng/mL or > 10.168 ng/mL and the 123 patients with mean tacrolimus trough levels between 4.609 ng/mL and 10.168 ng/mL (log-rank test, *p* = 0.009) (Figure 4). In an additional subgroup analysis, we found that a mean tacrolimus trough level between 4.431 ng/mL and 6.332 ng/mL for patients with a serum creatinine level > 1.311 mg/dL improved survival. Finally, this Cox’s model had a concordance of 0.904 and an adjusted generalized *R*^2^ of 0.388, indicating that it fit the survival data very well.

## 4. Discussion

This is the first study to report an association between long-term tacrolimus blood trough level and long-term patient survival in adult LT recipients. We found that a long-term tacrolimus blood trough level of 4.6–10.2 ng/mL in adult LT recipients is associated with a lower mortality rate. In patients with a serum creatinine > 1.3 mg/dL, a mean tacrolimus blood trough level of 4.4–6.3 ng/mL is associated with a lower mortality rate.

Decreasing tacrolimus use during long-term follow-up in LT recipients is a general concept in clinical practice. In this study, only one patient had the mean level > 10 ng/mL and a few patients had the mean level > 8 ng/mL (Figure 3). Patients with such high drug levels were most likely because of their poor compliance (not must because of they have truly such high trough level). Poor compliance indicates that a patient takes incorrect dosage of immunosuppressant that their physician prescribed, or takes immunosuppressant or blood exam at the wrong time, either of which might clearly be important factors that influence graft and survival outcome of LT patients during long-term follow-ups. We found that a long-term tacrolimus blood trough level of 4.6–10.2 ng/mL is associated with lower mortality rate; however, the upper limit of 10.2 ng/mL suggested by the regression analysis was merely a value for caution, but not a recommended upper limit in real clinical practice. The long-term hazard of high level of tacrolimus is already widely known; therefore, we would focus more on the findings that it appears to increase the survival risks in LT recipients if their tacrolimus levels are maintained below 4.6 ng/mL during the long-term follow-ups.

Inadequate immunosuppression is associated with higher risks of graft rejection [19], while excess immunosuppression increases the risks of malignancy and infection and increases the adverse effects of drug toxicity. The tailored use of immunosuppressants should be considered based on the patient’s risks of graft rejection and infection and the patient’s medical comorbidities and liver disease status prior to LT [14]. Patients with autoimmune liver diseases may require more immunosuppression to prevent disease recurrence and graft rejection [20]. By contrast, lower doses of immunosuppression are recommended in patients who have undergone LT due to HCV, as high levels of immunosuppression are related to increased viral replication [21,22]. Rejection results in abnormal liver function and is one of the most important factors associated with poor long-term graft and patient outcomes [23,24,25]. Tacrolimus reduces the risk of T-cell mediated rejection to protect graft function and is the cornerstone of a successful LT. However, short and long-term adverse effects of tacrolimus such as infection, chronic renal insufficiency, metabolic diseases (hyperlipidemia, hypertension, and diabetes mellitus), and malignancy have been widely reported and influence patients’’ long-term outcomes [7,8,9]. The early causes of death after LT within one year are infection and graft loss, and the late causes of death three years post-LT are malignancy, cardiovascular disease, and renal failure [10]. As graft function typically stabilizes, factors associated with the long-term outcome are often patient-related factors (such as chronic medical diseases) that are usually associated with tacrolimus.

Previous studies have reported that minimizing tacrolimus use in the early post-LT period is associated with a lower risk of new-onset diabetes mellitus [26], a lower incidence of hyperlipidemia [27], and better long-term survival [19,28]. In recent years, studies have focused on the reduction or complete withdrawal of long-term immunosuppressants in LT recipients [12,29,30,31,32,33,34]. One study reported satisfactory outcomes with the combined use of tacrolimus and mycophenolate mofetil, which allowed for the tacrolimus dose to be reduced [35]. Other studies demonstrated that the concomitant use of everolimus may reduce the required dose of tacrolimus while having potential renal benefits [29,36]. However, only some LT recipients were able to discontinue the use of immunosuppressants, and these patients more frequently experienced biopsy-proven acute rejection [29]. The risk of chronic rejection during long-term follow-up remains in patients with inadequate immunosuppression [30,33]. The tacrolimus blood concentration in LT recipients may play a key role in long-term outcomes, as it is associated with long-term graft function and its adverse effects are related to several chronic medical diseases. However, to the best of our knowledge, this is the first report regarding the effect of long-term tacrolimus levels on the long-term survival outcomes of LT recipients.

Several factors are associated with the long-term outcomes of adult LT recipients, including pre-transplant primary sclerosing cholangitis, immunosuppression therapies, acute and chronic rejections, malignancy, and metabolic syndrome [37,38]. As listed in Table 3, we found several independent risk factors of long-term mortality in this study. Only four patients aged <27 years at the time of LT were included in this study, and one died due to pneumonia 6.3 years after LT. Patients who underwent LT due to autoimmune liver diseases had worse outcomes, as they were more likely to experience acute rejections [39] and suffer disease recurrence, leading to graft loss. Patients who underwent LT due to HCV infections had relatively poor post-LT outcomes due to the disease recurrence followed by graft dysfunction and failure [40]. As direct-acting antiviral agents have advanced [41], the survival outcomes of HCV patients should improve in the near future. Long-term renal and liver functions are reflected by serum creatinine and total bilirubin levels, respectively. We found that abnormal serum creatinine levels have a bigger impact than abnormal serum total bilirubin levels on the survival outcomes of LT recipients. The graft function of adult LT recipients is chronically stable five years after LT. While some patients may experience asymptomatic hyperbilirubinemia at this time, the long-term outcomes remain favorable. By contrast, the gradual deterioration of renal function may lead to chronic kidney disease or end-stage renal disease, affecting the long-term survival, especially in patients with long-term use of tacrolimus.

This study had some limitations. First, the data were obtained from a single medical center in Asia, which provided a relatively small number of eligible patients with single ethnicity and fewer death events. Second, the study spanned a long time period, and therefore the improvements in surgical and medical expertise and advances in immunosuppression therapies may have influenced the patient outcomes. Third, we did not collect or analyze the time-dependent tacrolimus blood trough level during the follow-up visits five years after LT.

In summary, we found an association between the long-term tacrolimus blood trough levels and the long-term survival five years after LT. A mean tacrolimus blood trough level outside the range of 4.6–10.2 ng/mL appeared to be an independent risk factor for long-term mortality. Further studies with larger sample sizes are needed to verify these results and to further identify an appropriate tacrolimus blood trough level for maintenance use.

## Figures and Tables

**Table 1:** Univariate analysis for comparing the distributions of the demographic and clinical characteristics between the alive and dead adult liver recipients after five years of liver transplantations.

Variable	All Patients (*n* = 189)	Alive (*n* = 173)	Dead (*n* = 16)	*p* Value
Gender				0.7899
Male	121 (64.0)	110 (90.9)	11 (9.1)	
Female	68 (36.0)	63 (92.6)	5 (7.4)	
Age at LT (years)	52.7 ± 9.6	52.6 ± 9.5	53.4 ± 10.7	0.6672
Body weight at LT (kg)	64.9 ± 12.2	65.0 ± 12.3	63.7 ± 12.1	0.4459
Blood type				0.8719
O	79 (41.8)	73 (92.4)	6 (7.6)	
A	51 (27.0)	46 (90.2)	5 (9.8)	
B	42 (22.2)	39 (92.9)	3 (7.1)	
AB	17 (9.0)	15 (88.2)	2 (11.8)	
Graft type				1.0000
Living donor	144 (76.2)	132 (91.7)	12 (8.3)	
Deceased donor	45 (23.8)	41 (91.1)	4 (8.9)	
Etiology for LT				
Liver malignancy (HCC)	72 (38.1)	66 (91.7)	6 (8.3)	1.0000
Alcoholic cirrhosis	17 (9.0)	16 (94.1)	1 (5.9)	1.0000
HBV cirrhosis	100 (52.9)	92 (92.0)	8 (8.0)	1.0000
HCV cirrhosis	42 (22.2)	38 (90.5)	4 (9.5)	0.7577
Fulminant hepatitis	23 (12.2)	23 (100)	0 (0)	0.2259
Autoimmune disease	14 (7.4)	11 (78.6)	3 (21.4)	0.1017
Biliary atresia	5 (2.6)	5 (100)	0 (0)	1.0000
Other	10 (5.3)	9 (90)	1 (10)	0.5964
Total bilirubin (mg/dL)	0.973 ± 0.51	0.958 ± 1.53	1.133 ± 0.69	0.6260
Total bilirubin > 1 mg/dL	60 (31.7)	53 (88.3)	7 (11.7)	0.2770
Total bilirubin > 2 mg/dL	11 (5.8)	9 (81.8)	2 (18.2)	0.2360
Creatinine (mg/dL)	1.391 ± 1.25	1.279 ± 0.96	2.6 ± 2.74	0.0201 *
Creatinine > 1.5 mg/dL	31 (16.4)	23 (74.2)	8 (25.8)	0.0010 *
ESRD	5 (2.6)	2 (40.0)	3 (60.0)	0.0050 *
Tacrolimus mean level (ng/mL)	5.249 ± 1.71	5.263 ± 1.53	5.096 ± 3.12	0.9787
Tacrolimus level < 5 ng/mL	93 (49.2)	82 (88.2)	11 (11.8)	0.1216
Tacrolimus level < 4 ng/mL	44 (23.3)	36 (81.8)	8 (18.2)	0.0136 *
Tacrolimus level < 3 ng/mL	11 (5.8)	9 (81.8)	2 (18.2)	0.2356

Data are presented as mean ± standard deviation (SD) for continuous variables and frequency (percentage, %) for categorical variables. The *p*-values of statistical tests were calculated using the Wilcoxon rank-sum test for continuous variables and the Fisher’s exact test for categorical variables. * *p* value ≤ 0.05. Abbreviations: LT, liver transplantation; HCC, hepatocellular carcinoma; HBV, hepatitis B virus; HCV, hepatitis C virus; Total bilirubin, serum total bilirubin level; Creatinine, serum creatinine level; and ESRD, end-stage renal disease (defined by receiving hemodialysis regularly).

**Table 2:** The causes of 16 deaths since 5 years after adult liver transplantations.

Causes of Deaths	Number of Subjects
Malignancy	5 (31.25%)
De novo: Multiple myeloma, bladder cancer, colon cancer, prostate cancer	4
Recurrent: Hepatocellular carcinoma	1
Graft failure	4 (25.00%)
Chronic rejection	3
Autoimmune hepatitis	1
Infection	4 (25.00%)
Pneumonia	3
Urinary tract infection	1
Cerebral vascular event (intracerebral hemorrhage)	2 (12.50%)
Peptic ulcer bleeding	1 (6.25%)

**Table 3:** Multivariate analysis for identifying the predictors of long-term overall survival after 5 years of liver transplantations by fitting a multiple Cox’s Model in the adult liver transplant recipients ^1^.

Covariate ^2^	Estimate	Standard Error	Wald’s *z* Test	*p* Value	Hazard Ratio (HR)	95% Confidence Interval (C.I.)
Age at LT ≤ 27.011 years	5.1286	1.3872	3.6970	0.0002	168.7851	11.130–2559.512
Autoimmune (including PBC)	2.0946	0.7219	2.9015	0.0037	8.1221	1.973–33.431
HCV × Overall survival years	0.2924	0.0914	3.1978	0.0014	1.3397	1.120–1.603
Cre > 1.311 × T-Bil > 1.411 mg/dL	6.8262	1.5591	4.3784	<0.0001	921.6940	43.401–19,573.712
Cre > 1.311 × T-Bil ≤ 0.792 mg/dL	4.6604	1.3294	3.5056	0.0005	105.6778	7.805–1430.790
Cre ≤ 1.311 × T-Bil > 0.882 mg/dL	3.4174	1.1871	2.8788	0.0040	30.4913	2.977–312.341
Tacrolimus mean ≤ 4.609 or > 10.168 ng/mL	1.5599	0.6479	2.4076	0.0161	4.7581	1.336–16.940

^1^ The above multiple Cox’s model was fitted to the 189 adult patients who underwent liver transplantations with 16 death events, for modeling the hazard rate of the right-censored overall survival time five years after liver transplantations. All the cut-off values of the continuous covariates (e.g., age at liver transplantations) were estimated by choosing the option of applying the p-spline smoothing techniques in fitting simple and multiple Cox’s proportional hazards models (e.g., Figure 3). The time-dependent interaction term, HCV × Overall survival year, was added to the Cox’s model for handling the *non-proportional hazards* problem between the patients with and without HCV, and its positive-valued regression coefficient estimate, 0.2924, indicated that the risk of dying in the patients with HCV would increase as time elapsed five years after liver transplantations. Moreover, the three second-order interaction terms, Cre > 1.311 × T-Bil > 1.411, Cre > 1.311 × T-Bil ≤ 0.792, and Cre ≤ 1.311 × T-Bil > 0.882, were compared to the other two possible combinations, Cre > 1.311 × (0.792 < T-Bil ≤ 1.411) and Cre ≤ 1.311 × T-Bil ≤ 0.882, as the reference group (i.e., HR = 1.0), where the cross sign × can be literally interpreted as “and.” Finally, both goodness-of-fit (GOF) measures, *concordance* = 0.9041 (se = 0.0265) > 0.7 and *adjusted generalized R*^2^ = 0.3878 > 0.15, indicated an excellent fit of this multiple Cox’s model to the observed survival data. ^2^ Abbreviations: LT, liver transplantation; PBC, primary biliary cirrhosis; HCV, hepatitis C viral infection; Cre, serum creatinine level (mg/dL); T-Bil, serum total bilirubin level (mg/dL); and Tacrolimus mean, the averaged dosage of the tacrolimus-based immunosuppressant (ng/mL).

## Data Availability

The datasets used and analyzed during the current study are available from the corresponding author upon reasonable request.
